# Proteomics and its application in the research of acupuncture: An updated review

**DOI:** 10.1016/j.heliyon.2024.e33233

**Published:** 2024-06-18

**Authors:** Zhen Zhong, Meng-Meng Sun, Min He, Hai-Peng Huang, Guan-Yu Hu, Shi-Qi Ma, Hai-Zhu Zheng, Meng-Yuan Li, Lin Yao, De-Yu Cong, Hong-Feng Wang

**Affiliations:** a Changchun University of Chinese Medicine, No.1035 Boshuo Road, Jingyue National High Tech Industrial Development Zone, 130117, Changchun, China; b Department of Tuina, Traditional Chinese Medicine Hospital of Jilin Province, 130000, Changchun, China; c The Third Affiliated Hospital of Southern Medical University, No.183, West of Zhongshan Avenue, Tianhe District, Guangzhou, 510630, Guangdong Province, China

**Keywords:** Proteomics, Acupuncture, Electroacupuncture, Acupoints

## Abstract

As a complementary and alternative therapy, acupuncture is widely used in the prevention and treatment of various diseases. However, the understanding of the mechanism of acupuncture effects is still limited due to the lack of systematic biological validation. Notably, proteomics technologies in the field of acupuncture are rapidly evolving, and these advances are greatly contributing to the research of acupuncture. In this study, we review the progress of proteomics research in analyzing the molecular mechanisms of acupuncture for neurological disorders, pain, circulatory disorders, digestive disorders, and other diseases, with an in-depth discussion around acupoint prescription and acupuncture manipulation modalities. The study found that proteomics has great potential in understanding the mechanisms of acupuncture. This study will help explore the mechanisms of acupuncture from a proteomic perspective and provide information to support future clinical decisions.

## 1 Genral introduction of acupuncture with its holistic therapeutic theoreis

Acupuncture belongs to the category of complementary and alternative medicine, which originated in China more than 3000 years ago [1]. It began to spreadg to the America and Europe since the 16th century and since then it has attracted worldwide attention [2]. According to the traditional theories of meridians and acupoints in Classic Ancient Chinese Medical bookes (*e.g., HuangDi NeiJing*, also known as *The Yellow Emperor's Canon of Internal Medicin*e) [3], acupuncture therapies achieves the effects of treating various diseases by balancing the body's energy [4]. The Chinese medical theories believes that the whole body communicates and interacts between the internal organs and external body surface through meridians, along which are composed of many scattered functional areas so called acupoints [5,6]. Acupuncture needles of 25–50 mm in length and 0.25–0.45 mm in diameter are usually used to pierce the acupoints and produce soreness, numbness, and heaviness [6,7]. With the development of modern science and technologies, electrical stimulation has been extended to the traditional manual needling operation. Therefore, electroacupuncture (EA), which is widely used as a stimulation strategy, was also included in the scope of this review. Studies have shown that acupuncture has a high safety profile with few side effects [7].

The use of acupuncture for the treatment of various diseases has gained global recognition [8]. Clinical studies have shown that acupuncture has clear advantages especially in the treatment of pain [[9], [10], [11]], neurological disorders [12,13] and digestive disorders [14,15]. However, the mechanisms of effects associated with acupuncture are not fully understood, and further efficacy evaluations and validations by modern systems biology are still with challenges. Therefore, exploring biological mechanisms of acupuncture is a key issue that needs to be addressed. Acupuncture has been shown to regulate multiple biological processes and pathways from a “holistic view” with multi-level, multi-target, dynamic regulation [16]. In the last two decades, proteomics has been widely used to explore the mechanisms of acupuncture, owing to the advantages of high sensitivity, large scale, high throughput, and its similarity to acupuncture in terms of holistic and dynamic changes. Proteomics provides a powerful technical platform to explore the biological mechanisms of holistic regulation of acupuncture [17,18].

In this review, we will fist introduce the proteomics technique, then to briefly summarize the current applications and new outcomes of this technique in the acupuncture studies. Finally, we will discuss future research directions of acupuncture to raise caution that systematic and holistic detections and investigations should be focus for better exploring mechanisms of acupuncture therapies. This may conform more to the concept of the Chinese medicine theories from a “holistic view” with multi-level, multi-target, dynamic regulations.

## 2 The general development and category of the systematic proteomics technologies

Proteomics refers to the systematic study of the identity, variable abundance, distribution, modification, interaction, structure, and function of a large profile of proteins and their involvement in disease [19]. Since proteins are directly involved in the entire process of physiology, proteomics enables dynamic monitoring of changes in protein expression to clarify the underlying mechanisms of disease and further identify specific biomarkers as well as potential therapeutic targets [[20], [21], [22]].

Sample collection and preparation is a great starting point for obtaining valid information in the process of proteomics analysis [23]. In general, samples used for proteomics analysis can be divided into two categories: tissues (stomach, lungs, kidneys, brain, etc.) and biological fluids (plasma, serum, saliva, urine, tear fluid, cerebrospinal fluid, etc.) [24,25]. The more accepted methods of sample collection and preservation of tissues were the fresh-frozen method and formalin-fixed paraffin-embedded method [26]. There were also protocols and best practice tutorials for biological fluid sample collection [27,28]. However, there are still many challenges in the sample collection process. In the case of plasma, for example, differences in centrifugation methods, freezing temperatures, and time of blood collection can all have an impact on the results [29]. The complexity of the sample collection process should be given adequate attention.

**Fig. 1:**
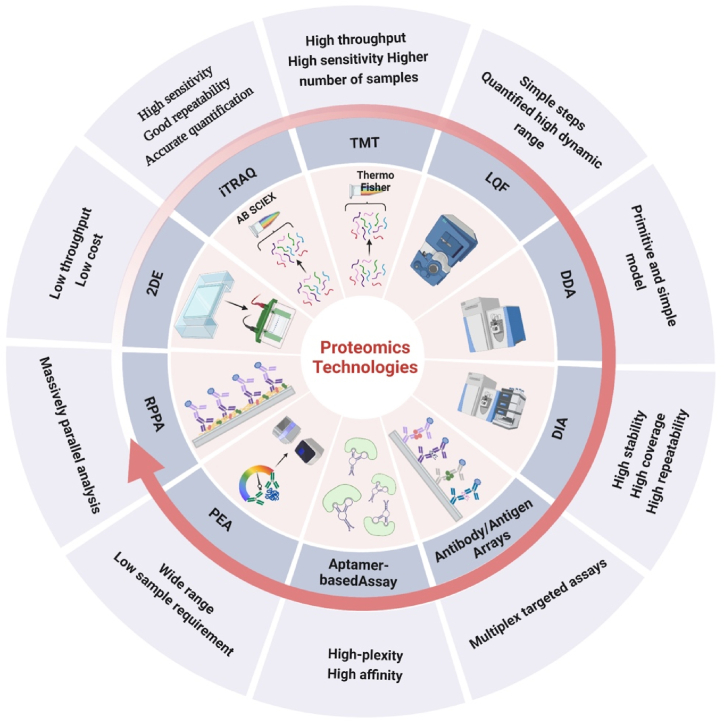
Overview of the main application features of proteomics technologies. The figure lists the current mainstream and innovative proteomics technologies. It also briefly describes the characteristics of each type of proteomics technology.

The development of proteomics depends to some extent on protein isolation techniques and the ability to identify and analyze proteins [30]. Currently, the most commonly used proteomics techniques include 2D electrophoresis (2-DE), mass spectrometry (MS), antibody/antigen arrays, etc. In the early discovery phase, protein screening is usually performed using low throughput methods such as 2-DE. This technique is still used today due to its robustness and low cost. The liquid chromatography-tandem mass spectrometry (LC-MS) has been increasingly chosen as the current core separation and identification for the proteomics research. Compared with 2-DE, LC-MS can be substantially more efficient, with advantages such as higher sensitivity and a larger detection range with lower injection samples [31]. With the advancement of technologies, various aspects of MS have been further optimized, especially the development of quantification techniques (*e.g*. isobaric tags for relative and absolute quantitation [iTRAQ], tandem mass tag [TMT], Label-free quantitation [LQF]), as well as the mass spectrometry scanning modes (data-dependent acquisition [DDA], data-independent acquisition [DIA]), etc. (Fig. 1) [32]. To meet clinical needs for easier detection and operation as well as faster imaging and recognization, many high-throughput and high-complexity protein chips technologies relying on the affinity reagents have emerged, such as antibody/antigen arrays, aptamer-based assays, proximity extension assay (PEA), reverse phase protein arrays (RPPA), etc [[33], [34], [35], [36]]. In addition, due to the high complexity of the human proteome, fractionation must be performed regardless of the analytical method used.

## 3 Application of proteomics in acupuncture research

**Fig. 2:**
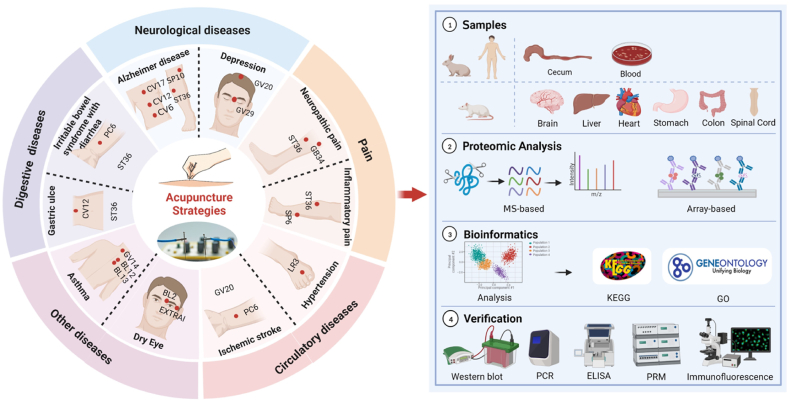
General research scheme of proteomics techniques acchieved in acupuncture studies. The inner ring (white) in the diagram on the left lists the main modalities of the acupuncture stimulation strategy (acupuncture and electroacupuncture). The outer ring is five color blocks corresponding to neurological, pain, circulatory, digestive, and other disorders. The middle circle is a common prescription of acupoints for major diseases. The figure on the right is a schematic diagram of the sample sources, proteomic methods, bioinformatics analysis, and validation of proteomics studies in acupuncture research.

Recently, proteomics technologies have been actively applied to the field of acupuncture to identify differentially expressed proteins (DEPs), to screen and discover biomarkers, and to further analyze the biological functions of DEPs that modulated by acupuncture therapies. These findings have helped clearify the efficacy and elucidate the mechanisms of acupuncture regulations. Fig. 2 depicts the general scheme of proteomics techniques that were acchieved in fields of acupuncture treatment. Proteomics techniques have been applied mainly in studies where the intervention modality is acupuncture and electro-acupuncture (EA), and a few studies have investigated the mechanism of action of acupuncture tonic and diaphoretic techniques. Body fluid (*e.g.,* blood, tears), and tissue organs (*e.g.,* brain tissue, spinal cord tissue, heart tissue, lung tissue, liver tissue, stomach tissue, etc.) from humans or animals after acupuncture interventions were used for proteomics studies. LC-MS and iTRAQ were the most commonly used proteomics techniques for acupuncture, followed by 2-DE and MALDI-TOF-MS, only several studies chose label-free and antibody array. For the bioinformatics annotation after the quantification, the Gene ontology (GO) and the Kyoto Encyclopedia of Genes and Genomes (KEGG) was the mostly used to assess the molecular function, cellular composition, and biological processes, as well as to determine the interaction between proteins and biological pathways. For the post experimental pathway validations, Western blot (WB), ELISA were further used to make the results more reliable. In addition, a few studies have explored protein-protein interaction networks in depth.

The overall effects of acupuncture include non-specific physiological effects and pathology-specific effects. Several studies have shown that acupuncture altered protein expression in humans and rats in healthy states produces [37,38]. It was suggested that the non-specific physiological changes of acupuncture were achieved through multiple biological pathways involving DEPs. However, more studies have explored the specific effects of acupuncture in disease states. Examples include acupuncture for neurological disorders, pain, circulatory disorders, and digestive disorders.

### 3.1 Proteomics studies in acupuncture against neurological disorders

Alzheimer's disease (AD) is a neurodegenerative disease that seriously endangers human brain memory and cognition [39], and imposes a huge economic burden on patients' families and society [40]. At present, there are no effective drugs that can curb the development of AD in clinical practice, only help with symptomatic relief [41]. Recent studies reported that acupuncture can help improve memory and cognitive impairment [42,43]. Table 1 summarizes the recent proteomics studies that provided scientific evidence for the acupuncture therapies for neurological disorders. SAMP8 mice have been widely used in studies of acupuncture for AD because of their markedly reduced learning and memory [44]. The biological effect produced by acupuncture of AD was related to alterations in protein expression. Hippocampus and amygdala were the main samples used for proteomic analysis. The composition of the acupuncture prescription has been key to the efficacy of acupuncture treatment. CV17, CV12, CV6, SP10, and ST36 were the commonly used acupuncture prescriptions, and BL23 and GV20 were often co-applied. The frequency of acupuncture is related to the specific disease and the animal model taken. Most studies chose to treat six times per week for a total of four weeks.

**Table 1:** Application of proteomics in acupuncture for neurological disorders.

Diseases	Therapy	Acupoints	Frequencies	Samples	Methods	DEPs	Functions and related pathways	Ref
AD	Acupuncture	CV17 CV12 CV6 SP10 ST36	3.5 min, 6/week, 4 weeks	SAMP8 mice; hippocampus	iTRAQ Labeling; LC-MS/MS;	605 DEPs (286 ↑, 319 ↓)	1. Acupuncture improved the learning, memory ability and regulates. 2. Synaptic and mitochondrial functions were enriched for regulating pathways.	[32]
	Acupuncture	CV17 CV12 CV6 SP10 ST36	3.5 min, 6/week, 4 weeks	SAMP8 mice; hippocampus	iTRAQ Labeling; LC-MS/MS; Immunofluorescence validation	605 DEPs (286 ↑, 319 ↓)	1. Acupuncture improved learning and memory ability. 2. Enrichment analysis showed that most of the proteins affected by needling were neuronal protrusions, the cytoskeleton, and involved biological processes such as intermediate filaments, keratin fibres, myelin sheaths, postsynaptic densities, and nerve fibre projections.	[33]
	Acupuncture	CV17 CV12 CV6 SP10 ST36	3.5 min, 6/week, 4 weeks	SAMP8 mice; hippocampus	iTRAQ Labeling; HPLC-MS/MS; Immunofluorescence and WB validation	158 DEPs	1. Acupuncture improved learning memory in the hippocampus. 2. Functional analysis showed that acupuncture could increase cytoskeleton-related proteins and “small G proteins” simultaneously.	[34]
	Acupuncture	CV17 CV 12 CV6 SP10 ST36	3.5 min, 6/week, 4 weeks	SAMP8 mice; hippocampal lipid rafts	HPLC-MS/MS	39DEPs	1. Acupuncture improved the cognitive ability. 2.3 cellular signaling pathways were involved: G protein-coupled receptor signaling, enzyme-linked receptor signaling, and ion channel-mediated signaling.	[35]
	Acupuncture	CV17 CV12 CV6 SP10 ST36	3.5 min, 6/week, 4 weeks	SAMP8 mice; hippocampus	iTRAQ Labeling; LC-MS/MS;	299 DEPs	1. Acupuncture significantly improved learning memory, and neuronal mitochondria were the key target. 2. KEGG results showed that oxidative phosphorylation, Parkinson's disease and protein processing in the endoplasmic reticulum are key pathways.	[38]
	Acupuncture	BL23 GV20 SP10 BL17	11 min, 6/week, 8 weeks	SAMP8 mice; hippocampus	2-DE; MALDI-TOF-MS; WB validation	13DEPs (9 ↑, 4 ↓)	1.Acupuncture modulated the expression of a variety of structural and functional proteins in hippocampal mitochondria for the treatment. 2. NFL, ATP-β, TBB2A and NDUS1 were validated.	[39]
	Acupuncture	GV20 BL23 BL17 SP10	11 min, 6/week, 8 weeks	SAMP8 mice; amygdala	2-DE; MALDI-TOF-MS	9DEPs (6 ↑, 3 ↓)	1.Acupuncture could improve mitochondrial energy metabolism, oxidative stress and reduce the production of Aβ to achieve the potential therapeutic effect.	[41]
	EA	GV20 BL23 GV16	20 min, 6/week, 16 weeks	APP/PS1 transgenic mice; Cerebral cortex and hippocampus	LQF; WB validation	319DEPs (149 ↑, 170 ↓)	1. EA prevented the decline of learning memory ability and the formation of Aβ age spots in the brain in a young rat model of AD. 2. KEGG analysis showed that the differential proteins were involved in alcohol addiction and the Apelin signaling pathway, among others.	[42]
VD	Acupuncture	ST36 GV20	20 min, 6/week, 2 weeks	Wistar rats; hippocampus	iTRAQ labeling; LC -MS/MS; WB validation	31DEPs	1. Acupuncture could improve cognitive function by reducing the production of reactive oxygen species and increasing the survival of neuronal cells. 2. GO analysis showed that most of the DEPs were related to oxidative stress, apoptosis and synaptic function.	[50]
PD	EA	GB34	20 min, 7/week, 2 weeks	C57BL/6 mice; SN	2-DE; MALDI-TOF MS; WB validation	22 DEPs (21 ↑, 1 ↓)	1. EA could protect DA neuron injury in mouse PD model. 2. CypA and NSF were selected as representative proteins for verification	[61]
	EA	GB34 GB39	20 min, 1/day, 12 days	C57BL/6 mice; striatum	2-DE; MALDI-TOF; WB validation	13DEPs (12 ↑, 1 ↓)	1. EA protected the cells of DA neurons. 2. HAGH was validated.	[62]
	EA	GV14 GV20	10 min, 6/week, 4 weeks	SD rats; the motor cortex	iTRAQ labeling; HPLC-MS; WB validation	95 DEPs (60 ↑, 35 ↓)	1. EA improved PD by attenuating dyskinesia in 6-OHDA rats. 2. KEGG pathway analysis showed that EA regulates AMPK signaling and adhesion pathways in impaired motor cortex.	[63]
Depression	EA	GV20 GV29	30 min, 7/week, 2 weeks	Wistar rats; PFC	iTRAQ labeling; HPLC-MS; WB validation	52DEPs	1. EA alleviated depression-like aphasic symptoms and anxiety behaviors, accompanied by improved synaptic morphology and an increase in PFC neurons. 2. KEGG pathway analysis identified seven pathways, including dopaminergic signaling, that contribute to the modulatory effects of EA.	[55]
	EA	GV20 GV29	30 min, 7/week, 1 weeks	Wistar rats; Hippocampus	iTRAQ; HPLC-MS/MS	274DEPs (145 ↑, 129 ↓)	1. EA improved antidepressant performance in depressed rats by protecting synaptic and mitochondrial functions in the hippocampus. 2. KEGG analysis revealed 5 important pathways including NAFLD, oxidative phosphorylation, Parkinson's disease, Alzheimer's disease, and Huntington's disease.	[57]
	EA	GV20 GV29	20 min, 7/week, 4 weeks	SPF rats; hippocampus	iTRAQ labeling; LC-MS/MS	27DEPs (14↑, 13 ↓)	1. EA improved depressive-like symptoms by regulating differential proteins. 2.27 proteins related to emotional disorders were successfully identified by iTRAQ.	[56]
Insomnia	Acupuncture	GV20 GV14 GV9	15 min, 1/day, 7 days	SD rats; hypothalamic	iTRAQ labeling; LC-MS/MS;	45 DEPs (28 ↑, 17 ↓)	1. Acupuncture could effectively improve sleep by regulating protein expression processes. 2. Enrichment of GO and KEGG pathways showed that prolamin, NMDA receptor synaptic nuclear signaling and neuronal migration factor, transmembrane protein 41B and microtubule-associated protein 1B were closely associated with neuromodulation or insomnia treatment.	[64]
Epilepsy	Acupuncture	HT8	6 min, 1/day, 3 days	C57BL/6 mice; hippocampus	2-DE; MALDI-TOF-MS; WB validation	11DEPs	1. Acupuncture altered the protein expression profile of the hippocampus, favoring neuronal survival in kainic acid-treated mice. 2. PURA and PP5 were selected for validation.	[65]
Maternal separation	Acupuncture	HT8	1min, 7/week, 1 weeks	SD rats; hypothalamic	2-DE; MALDI-TOF-MS	27DEPs (9 ↑, 5 ↓)	1. Acupuncture could induce severe stressful conditions in early life.	[66]

AD, Alzheimer disease; VD, Vascular dementia; PD, Parkinson's disease; DEPs, differentially expressed proteins; WB, Western blot; iTRAQ, isobaric tags for relative and absolute quantitation; LC–MS/MS, liquid chromatography with tandem mass spectrometry detection; HPLC, high performance liquid chromatography; 2DE, 2D electrophoresis; MALDI-TOF-MS, matrix-assisted laser desorption/ionization time of flight mass spectrometry; MSMS, tandem mass spectrometry; LQF,Label-free quantitation; GO, Gene ontology; KEGG, Kyoto Encyclopedia of Genes and Genomes; SD,Sprague-Dawley; SPF, specific pathogen free; PFC, Prefrontal cortex; SN, the substantia nigra; EA, electroacupuncture.

Several experiments reported that learning and memory abilities can be improved by acupuncture in animal models. Proteomic analysis showed that acupuncture substantially upregulated the expression of neuronal protrusion and cytoskeleton-related proteins, which underlie the structure and function of synaptic plasticity [45]. Another study achieved similar results and showed that DEPs were also involved in biological processes such as intermediate filaments, keratin fibers, myelin sheaths, postsynaptic density, and nerve fiber protrusions [46]. Further exploration of the neuronal cytoskeleton revealed that acupuncture at these points increased both cytoskeleton-associated proteins and small G proteins [47]. Nie et al. [48] reported that acupuncture recruited more kinases, ion channel proteins, and transmembrane signaling receptors, including G protein-coupled receptor-mediated pathways, which plays a key role in AD [49].

In addition, another key target for acupuncture in the treatment of AD was the neuronal mitochondria. Mitochondrial dysfunction was closely associated with the core pathological features of AD [50]. Acupuncture intervention in SAMP8 mice significantly upregulated the expression of mitochondria-related proteins [51]. Liang et al. [52] found that DEPs were involved in the regulation of mitochondrial function as well as structure after proteomic analysis. One of them, NFL, was one of the biomarkers of neurodegeneration and was closely associated with cognitive dysfunction [53]. Some researchers also found that the function of the differential protein involves oxidative stress and β-amyloid (Aβ) production in addition to mitochondrial energy metabolism [54]. Apart from SAMP8 mice, APP/PS1 double transgenic mice is another frequently used species for the AD study which expressing mouse/human amyloid precursor protein (Mo/HuAPP695swe) and mutant human progerin 1 (PS1-DE9). Zhang et al. [55] chosed the APP/PS1 juvenile mice to explore the potential mechanisms of acupuncture in the treatment of AD from a preventive perspective. This was known as “prevention before illness” in Chinese medicine theory. This study confirmed that EA was effective in preventing learning and memory deficits in APP/PS1 juvenile mice in adulthood and significantly reduced the formation of cortical and hippocampal Aβ age spots in AD model mice in adulthood. DEPs in the study such as epoxide hydrolase 4 (EH4), neurolysin, histone-H 3, GNB 5, Aβ, calsyntenin-3, myoglobin, metallothionein-1, and neurogranin have also been reported to be directly or indirectly associated with AD or Aβ [[56], [57], [58], [59], [60], [61]].

Vascular dementia (VD) is the second most common cognitive disorder after AD [62]. GV20 and ST36 have cerebral blood flow and anti-inflammatory effects in the ischemic area of the node [63]. Acupuncture ST36 and GV20 improved cognitive function in rats [64]. Proteomic analysis showed that S100B and SOD1 were differential proteins and the results were verified by Western blot to be reliable. Levels of S100B and SOD1 were considered to be markers of efficacy in subcortical vascular dementia [65,66]. The above studies suggest that proteomics is a useful tool to investigate the mechanisms underlying the effects of acupuncture prescription for AD. Through a variety of proteomic assays, it has been confirmed that acupuncture can modulate specific proteins and signaling pathways in multiple regions of the brain to improve cognitive levels in AD and VD.

Next, Parkinson's disease (PD) is also one of the predominant diseases treated with acupuncture [67]. The major behavioral impairment in PD was caused by a massive loss of dopaminergic (DA) substantia nigra (SN) neurons and depletion of striatal dopamine. GB34 was widely used to treat movement disorders because it protected SN and striatal DA neurons [68,69]. EA stimulation of GB34 protected nigrostriatal DA neurons in 1-methyl-4-phenyl-1,2,3,6-tetrahydropyridine (MPTP) mice [70]. Nine pinprick-specific proteins were found to be involved in cell death regulation, inflammation, or injury repair [71]. Proteomic analysis of the striatum of MPTP mice suggested that differential proteins may be involved in cellular metabolism. The protein HAGH was validated for reliable results. In addition, EA significantly improved spontaneous ground plane locomotion and stick-turning performance in a 6-hydroxydopamine (6-OHDA) unilateral injection-induced PD rat model [72]. EA-altered DEPs were involved in increasing autophagy, mRNA processing, and ATP binding as well as maintaining neurotransmitter homeostasis. In summary, EA facilitated the survival of DA neurons and attenuated toxicity such as oxidative stress, thus exerting a neuroprotective effect on different brain regions of PD mice.

Apart from the AD and PD, depression is another common neurological disorder [73]. EA has also been shown to be effective in the treatment of depression. In Chinese medicine, GV 20 and GV 29 are both part of the governor meridian, which reaches up to the medulla oblongata, so these two points are more effective in treating mental disorders. Therefore, these two acupoints are frequently used in combination for the treatment of depression [74,75]. Studies using samples from the prefrontal cortex have found that EA can alleviate symptoms of pleasure deficit, increase the number of PFC neurons and modulate dopaminergic signaling pathways to treat depression [76]. Analysis of the KEGG pathway revealed seven significantly enriched pathways in which the dopaminergic synapse, cocaine addiction, amphetamine addiction, and alcoholism pathways share the same four upregulated proteins. They were all important components of the dopaminergic synaptic signaling pathway. GUO et al. [77] also demonstrated that dopamine may be involved in the antidepressant-like effects of EA. In another study [78], EA treatment was also found to have an antidepressant effect by protecting mitochondrial function in the synapse and hippocampus. Further research is needed in the future to investigate the specific mechanism of action of EA in depression and whether the differential proteins identified are associated with EA or depression.

Moreover, acupuncture can also treat neurological disorders such as insomnia, epilepsy and maternal separation. Studies have shown that acupuncture treats neurological disorders by improving neuronal function, increasing neuronal survival, and promoting neurodevelopment [[79], [80], [81]].

### 3.2 Application of proteomics in acupuncture analgesia

In addition to the ability of acupuncture to treat neurological disorders, the effectiveness of acupuncture for analgesia has also been demonstrated [82,83]. The diseases in which proteomics techniques have been applied to study acupuncture analgesia include neuropathic pain (NP), inflammatory pain, and chronic pain (Table 2). Different types of pain involve various sites and mechanisms. Current samples applied for proteomic analysis include various regions of brain tissue, spinal cord, blood, etc.

**Table 2:** Application of proteomics in acupuncture analgesia.

Diseases	Therapy	Acupoints	Frequencies	Samples	Methods	DEPs	Functions and related pathways	Ref
NP	EA	ST36 GB34	30 min, 1/day, 12 days	Wistar rats; hippocampal	2-DE; MALDI-TOF MS; PCR and WB validation	19 DEPs (11 ↑, 8 ↓)	1. EA can reverse the reduction of thermal pain thresholds in the affected foot and plantar in the CCI model and had an analgesic effect on neuropathic pain. 2. Functional analysis revealed that DEPs are involved in cysteine metabolism, valine, leucine and isoleucine degradation and MAPK signaling.	[72]
	EA	ST36 GB34	30 min, 1/day, 12 days	Wistar rats; hypothalamus	2-DE; MALDI-TOF MS; PCR and WB validation	17 DEPs	1. EA was able to improve the affected foot and plantar thermal pain thresholds in the CCI model. 2. Functional analysis showed that changes in the expression of several proteins involved in REDOX enzyme activity, REDOX, protein binding, and glycolysis/gluconeogenesis/glucose metabolism may be involved.	[71]
	EA	ST36	30 min, 7/week, 1 weeks	SD rats; hypothalamus	2-DE; MALDI-TOF MS;	36 DEPs	1. EA had a relieving effect on mechanical pain. 2. DEPs were involved in inflammation, enzyme metabolism and signal transduction.	[73]
	EA	GB30 GB34	30 min, 7/week, 3 weeks	SD rats; hippocampal	TMT Labeling; LC-MS/MS; ELISA and WB validation	53 DEPs (17 ↑, 36 ↓)	1.EA improved neuropathic pain by eliminating mechanical pain sensitivity and memory deficits. 2. KEGG pathway analysis showed that DEPs were mainly enriched in drug metabolism, cytochrome P450 and isobiomass metabolism through cytochrome P450 pathway.	[74]
KOA	EA	ST36 GB34	30 min, 7/week, 2 weeks	SD rats; synovial	DIA-MS; WB validation	222 DEPs (144 ↑, 78 ↓)	1. EA could alleviate inflammatory pain behaviors and cartilage damage. 2. GO analysis suggested that cytokines secreted by macrophages may be involved in the regulation of KOA by EA.	[75]
DPN	EA	ST36 BL23	30 min, 4/week, 4 weeks	SD rats; The L4-5 spinal	TMT Labeling; LC-MS/MS;	97 DEPs (14 ↑, 83 ↓)	1. EA could improve DPN by regulating mechanical pain threshold and fasting blood glucose level in rats. 2. KEGG pathway enrichment analysis suggested that oxidative phosphorylation was a major factor involved in the effects of EA therapy on DPN.	[76]
BCP	EA	ST36 BL60	30 min, 7/week, 1 weeks	SD rats; the L4–L6 DRGs	PEX100 protein microarray (antibody array); WB validation	176 DEPs	1. EA alleviated mechanical pain behavior and to some extent bone destruction in BCP rats. 2. KEGG pathway analysis showed that ErbB signaling pathway, PI3K-Akt signaling pathway and MAPK signaling pathway were closely related to BCP or cancer.	[77]
pain aversion	EA	ST36 SP6	30 min, 7/week, 2 weeks	SD rats; The amygdala	iTRAQ labeling; LC-MS/MS; WB validation	11 DEPs	1. EA could increase the claw threshold of inflammatory pain, confirming the possible mechanism of EA in the central nervous system of pain aversion. 2. Glyceraldehyde-3-phosphate dehydrogenase, glutamate transporter-1 and P21-activated kinase 6 were selected for validation.	[78]
Inflammatory pain	EA	ST36 SP6	20 min, 1/day, 2 days	SD rats; The lumbar spinal cords	2-DE; MS/MS;	8 DEPs (4 ↑, 4 ↓)	1. EA stimulation attenuates CFA-induced inflammatory hyperalgesia. 2.8 loci were differentially phosphorylated proteins.	[79]
Chronic Myofascial Pain	Acupuncture	6 local twitch responses	20 min, 1/week, 4 weeks	SD rats; the gray matter	TMT Labeling; LC-MS/MS; PRM Validation	107 DEPs (47 ↑, 60 ↓)	1. Dry needling ameliorated chronic myofascial pain by modulating nociceptive mechanical thresholds in the left hind paw in an active myofascial trigger point rat model. 2. GO and KEGG enrichment results showed that dry needling can significantly modulate tightly connected pathways.	[80]
Chronic Pain	EA	LI10 LI11	30 min, 4/week, 3 weeks	SD rats; PFC and hippocampus	iTRAQ; LC-MS/MS; Immunoﬂuorescence and WB validation	8 DEPs(PFC); 14 DEPs(hippocampus)	1. EA could alleviate chronic pain syndrome, inhibit the cognitive dysfunction caused by chronic pain and restore normal cellular structure. 2. The MARKS in the PFC and PAK2 and ACAT1 in the hippocampus were further validated.	[81]
SCI	EA	GV6 GV9 T7 T11	20 min, 4/week, 3 weeks	SD rats; The spinal cord	2-DE; MALDI-TOF MS; Immunoﬂuorescence and WB validation	15 DEPs	1.EA could improve neuronal survival and thus contribute to SCI recovery. 2. ANXA5 and CRMP2 were further validated.	[82]
Migraine	Acupuncture	DU20 DU24 GB13 GB8 GB20	30 min, 3/week, 4 weeks	Human; Blood	DIA; HPLC-MS/MS	29 DEPs	1.Acupuncture could be effective in relieving migraines. 2. KEGG analysis showed differences in riboflavin metabolism and glycolytic pathways after acupuncture. This suggested that acupuncture modulation of migraine was associated with changes in energy metabolism.	[83]

NP, neuropathic pain; KOA, knee osteoarthritis; DPN, Diabetic Painful Neuropathy; BCP, Bone Cancer Pain; SCI, Spinal cord injury; WB, Western blot; TMT, tandem mass tag; DIA,indicates data-independent acquisition; PCR, polymerase chain reaction; ELISA, Enzyme-linked Immunosorbent Assay; PRM, Parallel reaction monitoring; DRGs, Dorsal root ganglions; CFA, Complete Freund's adjuvant.

In terms of the frequency of acupuncture, the frequency of treatment varied for the same animal models chosen. Each acupuncture treatment lasts 20–30 min. The treatment course is usually higher in SD rats than in Wistar rats. In terms of acupoint selection, ST36 and GB34 are reported to be the key acupoints for analgesia in Chinese medicine, the ability of which by traditional needling stimulation have been reported by recent laboratory studies [84,85]. EA interventions with ST36 and GB34 were reported to reverse the reduction in thermal pain thresholds in the affected foot and plantar surface of chronic constrictive injury (CCI) rats [86,87]. Proteomic analysis of the hippocampus and hypothalamus using a 2-DE combined with MALDI-TOF MS strategy showed that EA analgesia may be achieved through the regulation of multiple protein expression as well as signaling pathways, including inflammation, enzyme metabolism and signal transduction. Similar results were obtained in a study by Sung et al. [88] Notably, Gong et al. [89] chose GB34 combined with GB30 by EA to intervene in the hippocampus of NP mice, reporting that hippocampal TMEM126A plays an important anti-inflammatory role in EA for neuropathic pain. Furthermore, pain caused by other diseases, such as osteoarthritis of the knee (KOA), diabetic painful neuropathy (DPN), and bone cancer pain (BCP), could also be treated with EA on the acupoints of ST36 and GB34 [[90], [91], [92]]. These studies revealed by different proteomic analysis techniques that EA could alleviate inflammatory pain behavior and cartilage damage, could inhibit cytokines secreted by macrophages in KOA, could regulate oxidative phosphorylation in DPN disease, and could alleviate pain and bone destruction in BCP disease. Among them, Wang et al. [93] found that the potential mechanism of EA treatment for BCP by PEX100 protein microarray may be related to mTOR phosphorylation and mTOR pathway.

ST36 and SP6 are another common pairs of acupoint prescriptions for analgesia, especially for inflammatory pain. Several studies have demonstrated that EA interventions on ST36 and SP6 reduced thermal nociceptive hypersensitivity in rats in a complete Freund's adjuvant (CFA)-induced inflammatory pain model [94,95]. On the one hand, a study performed and validated proteomic analysis of the amygdala, showing that the expression of GAPDH, GLT-1, and PAK6 was closely associated with EA intervention in this disease. On the other hand, the spinal cord samples was collected for phosphoproteomic analysis, and a total of eight differentially phosphorylated proteins were identified (Table 2). These proteins are involved in processes such as cellular signaling, protein transport, and transcription. In summary, both central and peripheral mechanisms of acupuncture for inflammatory pain have been initially confirmed. Valuable protein biomarkers for acupuncture analgesia were provided.

Chronic pain is widespread across the globe. Several studies have shown that acupuncture helps to modulate the mechanical threshold of nociception and alleviate chronic pain syndromes [96,97]. Li et al. used acupuncture to intervene in the gray matter of chronic fascial pain mice for proteomic analysis and validated Actin α3, Calsequestrin-1, and microalbumin α by parallel reaction monitoring (PRM). Studies have confirmed the dominant role of the tight junction pathway in the management of chronic pain with acupuncture. Another study showed that in addition to relieving chronic pain, acupuncture can inhibit chronic pain-induced cognitive dysfunction and restore normal cellular structure. Proteomic analysis of the PFC and hippocampus revealed that marcks, pak2, and acat1 may be potential targets for acupuncture treatment. This indicates that the central mechanism of acupuncture to improve pain involves multiple brain regions, further demonstrating that acupuncture is a holistic, multi-targeted treatment modality.

In addition, Li et al. [98] investigated the effect of EA on spinal cord injury (SCI) and demonstrated that EA is useful for neuronal survival. Proteomic analysis identified ANXA 5 and CRMP 2 as key pinprick-specific proteins. In another study, Liu et al. [99] selected human blood as a sample to investigate the mechanism of acupuncture intervention in migraine. The study demonstrated that acupuncture can relieve migraine by regulating energy metabolic pathways.

### 3.3 Application of proteomics in acupuncture for circulatory diseases

This section evaluates the proteomic changes associated with acupuncture in both human and animal models of hypertension and ischemic stroke (IS) to investigate the potential mechanisms through which acupuncture can alleviate circulatory disorders. Table 3 provided comprehensive details regarding the specific acupuncture methods utilized, the acupoints selected, the samples, and the proteomics methodologies employed in this section.

**Table 3:** Application of proteomics in acupuncture for circulatory diseases.

Diseases	Therapy	Acupoints	Frequencies	Samples	Methods	DEPs	Functions and related pathways	Ref
Hypertension	acupuncture	LR3	5 min, 7/week, 1 weeks	SD rats; The medulla	2-DE; MALDI-TOF MS; qRT-PCR, WB and ELISA validation	23 DEPs	1. Acupuncture could significantly reduce systolic blood pressure in rats, but not enough to reduce blood pressure to normal level. 2. Reduced oxidative stress may be a potential mechanism for acupuncture in the treatment of hypertension.	[89]
	EA; TRFM; TRDM	LR3	20 min, 7/week, 2 weeks	SHR and WKY rats; hypothalamus	Label-free; LC-MS; PRM-MS validation	117 (EA/M), 61 (TRFM/M) and 86 (TRDM/M) DEPs	1. TRDM, TRFM and EA procedures reduced blood pressure measurements, with TRDM being the most effective of the techniques used. 2.12(RF/M),11(RD/M),15(EA/M) pathways were significantly different in terms of protein enrichment, respectively.	[86]
	TRFM; TRDM	LR3	20 min, 7/week, 2 weeks	SHR and WKY rats; the parietal cortex	Label-free; LC-MS	72 DEPs in TRFM/M 1043 DEPs in TRDM/M	1. Acupuncture had a blood pressure regulating effect, and the TRDM group had a better antihypertensive effect than the TRFM group. 2. TRDM improved oxidative phosphorylation, regulates vasoconstriction and smooth muscle proliferation, improves lipid metabolism and reduces glucose metabolism, improves inflammation and endothelial function, and reduces sympathetic excitability.	[87]
	RF; RD; EA	LR3	20 min, 7/week, 2 weeks	SHR and WKY rats; Cerebellum	Label-free; LC-MS; PRM-MS validation	96,133 and 216 DEPs	1.RD, RF, and EA at LR3 lowered blood pressure, with RD being the most effective. 2. KEGG pathway enrichment indicated that there were significant differences in protein enrichment in 12 (RF/M), 11 (RD/M) and 15 (EA/M) pathways respectively.	[88]
IS	EA	GV20 GV24	30 min, 7/week, 2 weeks	SPF rats; hippocampal	TMT labeling; LC -MS/MS; WB validation	218DEPs (168 ↑, 50 ↓)	1.EA significantly alleviated neurological deficits, spatial learning and memory deficits, and cerebral infarction in cerebral ischemic stroke rats. 2. GO enrichment analysis showed that DEPs were associated with brain and neurodevelopment.	[92]
	EA	MS6 BL10 GB20 LI4 PC6 B40 SP6 ST36	30 min, 1/day, 10 days	Human; Blood	2-DE; MALDI-TOF/TOF-MS; WB validation	7 DEPs (6 ↑, 1 ↓)	1.EA could improve the muscle strength of the upper and lower limbs to treat acute IS. 2.7 DEPs were found. SerpinG1, coagulation proteins and C3 were validated.	[93]
	Acupuncture	GV20 2 points beside GV20	30 min, 6/week, 2 weeks	SD rats; the brain	SDS-PAGE; LC-MS/MS;	414 DEPs	1. Acupuncture improved neurological function scores and motor dysfunction in a middle cerebral artery occlusion model rat. 2. DEPs were mainly involved in cellular signal transduction, protein transport, and exerted their biological functions through various synaptic and metabolic pathways.	[99]
I/R injury of the heart	EA	PC6	30 min, 1/week, 1 weeks	SD rats; heart tissue	2-DE; Antibody and WB validation	26DEPs	1. EA reduced I/R injury in rat heart. 2. The expression of myocardial interleukin-1 B-converting enzyme (ICE) was gradually decreased by EA treatment, while the expression of glycogen synthase-3 a (GSK-3) was increased in EA.	[102]

IS, ischemic stroke; I/R, Ischemia-reperfusion; WB, Western blot; SDS-PAGE, sodium dodecyl sulphate-polyacrylamide gel electrophoresis; qRT-PCR, Quantitative Reverse Transcription-polymerase Chain Reaction; SHR, spontaneously hypertensive rats; WKY, Wistar-Kyoto; TRFM, twirling reinforcing manipulation; TRDM, twirling reducing manipulation; RF,twirling reinforcing manipulation group; RD,twirling reducing manipulation group.

Acupuncture has been shown to be an effective treatment of hypertension [100]. Previous research has established that hypertension pathogenesis is linked to the central nervous system's malfunctioning brain regions [101]. Proteomics studies have confirmed the effectiveness of acupuncture in treating hypertension [[102], [103], [104], [105]]. Four studies have used acupuncture to intervene on LR3 in spontaneously hypertensive rats (SHRs) and found more than 100,000 DEPs in various brain regions, including the medulla oblongata, hypothalamus, parietal cortex, and cerebellum (refer to Table 3). The majority of DEPs underwent validation through parallel reaction monitoring (PRM), and the results were found to be generally consistent with the experimental trends observed. Early studies have found that acupuncture on LR3 relieves hypertension compared to non-acupoints [105]. Proteomics studies have shown that needling LR3 can alter the protein expression profile of SHRs, and the regulatory mechanisms involve multiple biological processes in multiple brain regions. Out of these findings, the neoplastic neurotrophic factor (NENF) has been suggested as a possible target for hypertension treatment [102]. Mechanisms frequently detected by acupuncture antihypertensive proteomics primarily include improved oxidative phosphorylation and regulation of vasoconstriction and smooth muscle proliferation [103,104].

Different methods of acupuncture manipulation were one of the influencing factors that produced the aforementioned differential expression of proteins and biological functions. The Twirling reducing manipulation (TRDM) and the Twirling reinforcing manipulation (TRFM) are both acupuncture manipulation methods that achieve augmentation and decrementation by manually twisting the needle. Interestingly, these studies compared also the impact ofthe TRDM with the TRFM on managing hypertension, reported that TRDM was the most effective in lowering the blood pressure [[102], [103], [104]]. In the future, it would be worthwhile to explore the hypotensive effects of specific acupuncture operations on various acupoints. A further selection of other brain regions for proteomic analysis will help to systematically reveal the potential mechanisms of action of different acupuncture manipulations.

IS is a circulatory disease characterized by high mortality and self-injury rates [106]. Acupuncture can significantly improve the motor dysfunction and cognitive level caused by stroke [107]. Recently, researchers have been actively investigating reliable biomarkers for IS, which can help identify effective targets and mechanisms for acupuncture treatment of IS. Notably, differential expression of various proteins, including Pak4, Akt3, Efnb2, SerpinG1, complement component I, C3, C4B, and beta-2-glycoprotein I, were observed after acupuncture treatment for IS [108,109]. In particular, Pak4, Akt3, and Efnb2 are considered to be important neuroprotective factors [[110], [111], [112]]. Complement components and beta-2-glycoprotein I also play an important role in the pathogenesis of IS [113,114]. Thus, it was hypothesized that the neuroprotective effects of acupuncture for IS may be mediated through mechanisms such as complementary activation, inhibition of apoptosis, and anti-thrombosis. Other reports also showed that acupuncture increased the expression levels of Cdc42 and GFAP in rats with middle cerebral artery obstruction [115]. GFAP can be used to identify stroke subtypes, which can be extremely helpful for acupuncture precision treatment [116,117]. Additionally, acupuncture can play a cardioprotective role against I/R injury by regulating cytochrome P450 and glycogen synthase kinase-3 a [118].

### 3.4 Application of proteomics in acupuncture for digestive system diseases

According to Chinese medicine principle, therapeutic effects may differ when using different combinations with acupoints. Therefore, identifying a scientific and reasonable effective combination of acupoints is crucial for improving acupuncture efficacy. There are some commonly used acupoint combinations for treating digestive system diseases by acupuncture [119,120]. For example, ST36-CV12 and ST36-PC6 have been applied to ameliorate gastrointestinal disorders (Table 4) [[121], [122], [123], [124], [125]].

**Table 4:** Application of proteomics in acupuncture for digestive system diseases.

Diseases	Therapy	Acupoints	Frequencies	Samples	Methods	DEPs	Findings	Ref
Stress gastric ulcer	Acupuncture	CV12 ST36	10 min, 7/week, 1 weeks	Wistar rats; Stomach tissues	SDS-PAGE; nano-LC/MS	14 DEPs	1.The combination of CV12 and ST36 was superior to single acupoints and other acupoints combinations for the prevention of stress ulcers. 2. DEPs species included enzymes, backbone proteins, transport proteins and heat shock proteins.	[105]
gastric ulcer	EA	CV12 ST36	20 min, 7/week, 1 weeks	SD rats; Stomach tissues	TMT labeling; HPLC-MS; ELISA; WB validation	133 DEPs (55 ↑, 78 ↓)	1. Stimulation of CV12 and ST36 by EA improved gastric ulcers. 2. KEGG results showed that the gastric acid secretion pathway was significantly regulated after acupuncture.	[106]
CAG	acupuncture	RN12 PC6 ST36	20 min, 3/week, 20 weeks	Human; peripheral blood	iTRAQ Labeling; HPLC-MS/MS; ELISA validation	66 DEPs	1. Acupuncture could improve the histopathological changes of gastric mucosa in CAG patients. 2. Bioinformatics analysis showed that actin binding proteins (ABPs) and Notch signaling pathway related proteins were closely related to the occurrence and development of CAG.	[107]
IBS-D	Acupuncture	ST36 PC6 CV4	18 min, 6/week, 4 weeks	SD rats; colon	LQF; LC-MS/MS; WB validation	56 DEPs (37 ↑, 19 ↓)	1. Acupuncture could alleviate IBS-D. 2. IBS-D was related to processes such as energy metabolism and muscle excitation/contraction, and acupuncture could reverse the impaired normal energy metabolism.	[108]
Enteritis	EA	ST36	30min, 2/week, 3weeks	New Zealand white rabbits; The PVS on the cecum	TMT; LC-MS/MS; WB validation	110 DEPs (65 ↑, 45 ↓)	1. EA could relieve bleeding, ulcers, adhesions and thickening of the intestinal wall of enteritis and regulate the inflammatory process. 2. Enrichment analysis revealed possible involvement in the treatment of enterocolitis through processes such as promotion of inflammatory cell proliferation, antigen expression and cell adhesion.	[110]
T2DM with NAFLD	acupuncture	BL13 BL20 BL23 LI4 ST36 SP6 LR3	20 min, 6/week, 2 weeks	db/db and db/m mice; liver tissues	TMT/iTRAQ labelling; HPLC-MS/MS; ELISA; PRM validation	122 DEPs (73 ↑, 49 ↓)	1. Acupuncture could reduce the risk of NAFLD by reducing lipid storage in liver cells, inhibiting de novo fat synthesis, and promoting fatty acid oxidation. 2. DEPs of KEGG pathway are mainly involved in PPAR signaling pathway, fatty acid biosynthesis, fatty acid metabolism, fatty acid elongation, fat digestion and absorption.	[109]

CAG, chronic atrophic gastritis; IBS-D, Irritable bowel syndrome with diarrhea; T2DM, Type 2 diabetes mellitus; NAFLD, non-alcoholic fatty liver disease; PVS,primo vascular system; DA,attenuates dopaminergic; WB, Western blot.

The acupuncture treatment with acupoint combination of ST36-CV12 on gastric ulcer rats model displayed 14 relevant DEPs, mainly including enzymes, motor (skeletal) proteins, transporter proteins, and heat shock proteins [121]. The same combination of acupoints using EA on a gastric ulcer rats model evaluated proteomic changes and found 133 DEPs in another study by the same research team, which may be due to different proteomic techniques used [122]. These studies suggested that acupuncture improves gastric ulcer symptoms by regulating abnormal gastric acid secretion, stimulating the immune system, positively regulating apoptosis, and promoting the ability to repair gastric mucosal damage. Another report also assessed proteomic changes in the peripheral blood serum of patients with acupuncture intervention in chronic atrophic gastritis (CAG) [123]. In addition to ST36-PC6, RN12 was added to the selection of acupoints. Bioinformatic analysis speculated that acupuncture achieved the treatment of CAG by modulating actin-binding proteins and gap-signaling pathway-related proteins. In conclusion, the above studies indicated that interfering with different combinations of acupoints can lead to different protein expression and pathways. The proteomic study of acupuncture with multiple acupoint combinations deserves further in-depth investigation.

In irritable bowel syndrome (IBS-D), proteomic analysis has shown an association with biological processes such as energy metabolism and muscle excitation/contraction [124]. Acupuncture reversed the impairment of normal energy metabolism and used Atp5a1 and Bpnt1 as key targets. Moreover, TMT-based proteomic analysis was utilized to identify changes in protein expression in rabbit models of enteritis treated by EA, revealing that most of the identified DEPs were related to inflammatory and immune processes [126]. Among them, CD40, CD45, HLA-DRA1, LAMP1, JAGN1 and FGL1 were alleviated or reversed by EA treatment, showing that EA has significant anti-inflammatory effects.

In a study of acupuncture for the treatment of type 2 diabetes mellitus (T2DM) combined with nonalcoholic fatty liver disease (NAFLD), liver tissue from mice was analyzed using proteomics [125]. KEGG analysis revealed that DEPs were mainly enriched in the PPAR signaling pathway, fatty acid biosynthesis, fatty acid metabolism, fatty acid elongation, fat digestion, and absorption. Acupuncture was found to reduce the risk of hepatocyte steatosis by inhibiting lipid resynthesis, promoting fatty acid oxidation, and regulating the expression of proteins related to glucose and lipid metabolism. These findings may provide new targets and pathways for acupuncture to treat digestive disorders. In addition the frequency of acupuncture for digestive disorders is generally low compared to other systemic disorders. Each treatment typically lasts 10–30 min. The overall course of treatment is 1–4 weeks. Except for CAG, of course, which is related to the fact that the samples are human blood and the characteristics of the disease.

### 3.5 Application of proteomics in acupuncture for other diseases

In addition to the diseases mentioned above, proteomics has been applied in the field of acupuncture for treating respiratory diseases, ophthalmic diseases, immune system diseases, gynecological diseases, etc. Acupoints GV14, BL12, and BL13 are frequently used in acupuncture treatment for Asthma, which is a chronic inflammatory disease of the respiratory tract that has a wide impact worldwide [127]. A proteomic study showed that acupuncture reduced the expression of SLC3A2, ATP1A3, S100A8, RAGE, and S100A11 and increased the expression of CC10, ANXA5, sRAGE, and Cyp A in lung tissues and serum of an Obamacin-induced mouse asthma model [[128], [129], [130]]. These results suggested that acupuncture could treat asthma by regulating processes such as iron sagging, oxidative stress, immune regulation, gene expression, and protein synthesis.

In ophthalmic diseases, acupuncture can improve ocular surface disease index scores and symptoms in patients with dry eye disease (DED) by intervening at common acupuncture points such as BL2,EXTRAI and stimulating lacrimal gland secretion. In a proteomic study on the effect of acupuncture on tear secretion in rabbits, six proteins were upregulated while five were downregulated [131]. Of these, Annexin A1 and S100A9 were considered as potential therapeutic targets for DED [132]. Another study showed a twofold increase in tear protein counts of secreted proteins and a significant decrease in cytosolic proteins after acupuncture [133]. Tong L et al. [134] found similar results.

Gynecological disorders, such as ovarian hyperstimulation syndrome (OHSS), can also be effectively treated by acupuncture. A proteomics study reported that the EA therapy could reduce oocyte numbers and preserve the vascular barrier against OHSS by triggering a CD200-mediated anti-inflammatory response, and could reverse the down-regulation of CD200 in the ovaries of the OHSS rat model [135]. Overall, this study provided a scientific basis for the potential targets of acupuncture for OHSS.

## 4 Proteomic characterization of acupuncture mechanisms

**Fig. 3:**
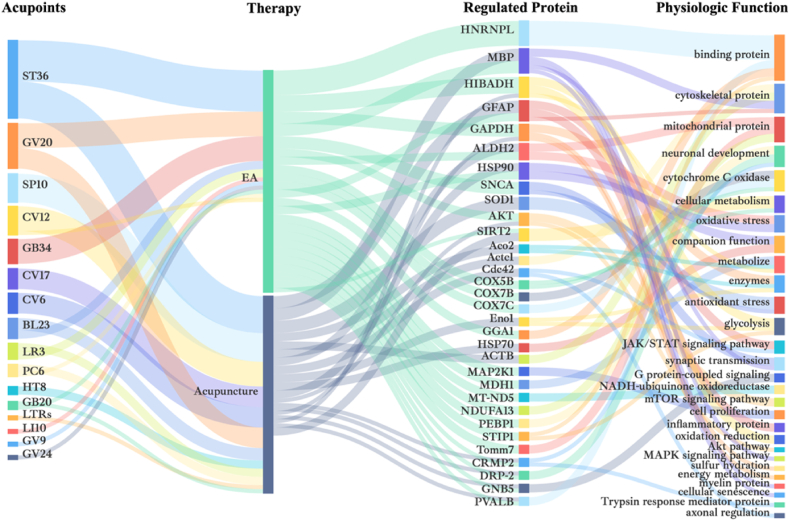
Proteomic characterization of acupuncture mechanisms. The diagram summarizes the overlapping acupoints, therapy, regulated proteins, and phsiologic functions.

In summary, all proteomics studies in the field of acupuncture have provided a large number of proteins that may be involved in the mechanism of disease amelioration by acupuncture. Most acupuncture regulation of proteins may be specific to each disease type or sample tissue collected. However, a deeper comparison of the above proteomics studies applied to acupuncture reveals a small amount of overlapping protein regulation (Fig. 3). Interestingly, some of the proteins were regulated in multiple diseases as well as in different modalities of acupuncture intervention. Overall, the higher frequencies in the selection of acupuncture points were ST36,SP10,CV12,GV20 and GB34.

Based on the physiological functions of the proteins, the overlapping proteins (in order of overlap frequency) can be broadly categorized as binding proteins, cytoskeletal proteins, mitochondrial proteins, neuronal development-related proteins, cytochrome C oxidase, cellular metabolism, oxidative stress, companion function, metabolism, enzymes, and antioxidative stress-related proteins. These regulated proteins are mainly involved in inflammation, apoptosis, neuronal damage repair, mitochondrial function, signaling, energy metabolism, and other processes. The major pathways involved include glycolysis, JAK/STAT signaling pathway, mTOR signaling pathway, and MAPK signaling pathway.

Of these, HNRNPL, MBP and HIBADH were all present in pain [136,137]. HIBADH is strongly correlated with oxidoreductase activity and redox [138,139]. And the process of acupuncture analgesia involved ameliorating oxidative damage to the cytoskeleton, as well as energy exchange [140]. Acupuncture reduced the expression of HNRNPL, regulated the stability of coupling proteins, and affected neuronal activity, resulting in analgesic effects. MBP not only can relieve pain by participating in the regulation of MAPK signaling pathway, but also widely found in neurological diseases, circulatory diseases [17]. MBP is associated with synapse-associated myelin formation, and its expression has been linked to neuronal cell injury. Acupuncture was able to regulate MBP levels, improve neurodegenerative lesions, and protect ischemic brain tissue. In conclusion, acupuncture is able to ameliorate diseases by regulating multiple proteins and participating in multiple pathways. It is of great interest to apply proteomics techniques to explore the mechanism of action of acupuncture.

## 5 Concluding remarks

Acupuncture is an integrative medical therapy, which has great potential in the prevention and treatment of various diseases. Proteomics-based approaches and bioinformatics annotation can help identify potential biomarkers and provide biological intepretations of potential mechanisms for acupuncture treatments. According to published proteomic studies to date, acupuncture could modulate a large number of key proteins in multiple systems and organs. However, the specific mechanisms by which acupuncture interacts with these different systems have not been clearly elucidated. First, in terms of sample selection, acupuncture has not been well studied on body fluids, involving only blood and tears. The process protocol for sample collection was also not kept up to date. Sample selection is directly related to the needs of clinical research. Only by expanding the space for sample selection and standardizing the process of sample collection can we cover the scope of acupuncture in treating more diseases. Second, the use of proteomics technology was generally less advanced in the field of acupuncture. Only one study used antibody arrays. The quality of bioinformatics analyses that follow proteomics was variable due to the long span of years. The lack of a validation process in some studies reduced the credibility of the results. In addition, most of the existing studies were based on disease states, and there were fewer studies applying proteomics technology to explore the specificity of acupuncture points in physiological states. There were insufficient comparative studies on the frequency of acupuncture, manipulation, and prescription of different acupoints.

Therefore, in the future, consideration should be given to expanding the selection of diseases and conducting systematic studies by combining metabolomic, transcriptomic, and other multi-omics techniques in order to elucidate the scientific basis for the efficacy of acupuncture. Further in-depth exploration from a broader and more systematic perspective using advanced proteomics technologies that are more targeted, sensitive and reproducible. To strengthen the research on acupuncture techniques and acupuncture parameters with a view to applying proteomics technology to standardize the therapeutic criteria for acupuncture treatment of different diseases. In addition to acupuncture and EA, other stimulation strategies, such as cupping and acupuncture piercing, have also been studied by applying proteological techniques [[141], [142], [143]]. In conclusion, proteomics-based acupuncture studies are expected to complement new discoveries in acupuncture modernization and thus deepen the understanding of the mechanisms of acupuncture action. And ultimately improve the patient's disease state at the individual level.

## Funding

This study was supported by the 10.13039/501100001809National Natural Science Foundation of China (grant number. 81874502); National Key Research and Development Program (grant number. 2018YFC1706000); 10.13039/501100003807Jilin Province Science and Technology Development Project (grant number. YDZJ202301ZYTS165).

## Data availability statement

This research was not applicable. No data was used for the research described in the article.

## CRediT authorship contribution statement

**Zhen Zhong:** Conceptualization, Visualization, Writing – original draft. **Meng-Meng Sun:** Project administration. **Min He:** Project administration. **Hai-Peng Huang:** Supervision. **Guan-Yu Hu:** Supervision. **Shi-Qi Ma:** Investigation, Formal analysis. **Hai-Zhu Zheng:** Formal analysis, Investigation. **Meng-Yuan Li:** Data curation, Methodology. **Lin Yao:** Data curation, Methodology. **De-Yu Cong:** Funding acquisition, Writing – review & editing. **Hong-Feng Wang:** Funding acquisition, Writing – review & editing.

## Declaration of competing interest

The authors declare that they have no known competing financial interests or personal relationships that could have appeared to influence the work reported in this paper.

## Abbreviations

2DE: 2D electrophoresis
DEPs: differentially expressed proteins
DIA: indicates data-independent acquisition
EA: electroacupuncture
ELISA: Enzyme-linked Immunosorbent Assay
GO: Gene ontology
HPLC: high performance liquid chromatography
iTRAQ: isobaric tags for relative and absolute quantitation
KEGG: Kyoto Encyclopedia of Genes and Genomes
LC–MS/MS: liquid chromatography with tandem mass spectrometry detection
LQF: Label-free quantitation
MALDI-TOF-MS: matrix-assisted laser desorption/ionization time of flight mass spectrometry; MSMS, tandem mass spectrometry
PCR: polymerase chain reaction
PRM: Parallel reaction monitoring
SDS-PAGE: sodium dodecyl sulphate-polyacrylamide gel electrophoresis
TMT: tandem mass tag
TRDM: twirling reducing manipulation
TRFM: twirling reinforcing manipulation
